# How to Avoid Coronary Occlusion During TAVR Valve-in-Valve Procedures

**DOI:** 10.3389/fcvm.2019.00168

**Published:** 2019-11-19

**Authors:** Roberto Valvo, Giuliano Costa, Marco Barbanti

**Affiliations:** Division of Cardiology, A.O.U. Policlinico—Vittorio Emanuele Hospital, University of Catania, Catania, Italy

**Keywords:** TAVR, valve-in-valve, coronary occlusion, coronary protection, BASILICA

## Abstract

Transcatheter aortic valve-in-valve replacement has been recently reported as a less-invasive alternative to re-do surgery in patients with bioprosthetic valve failure. Although procedural success is achieved in the great majority of patients, this therapy is associated with several potential complications, and coronary occlusion is one of the most feared. This is a rare event, but it is associated with an extremely poor prognosis. In this review, the mechanisms, the identification of patients at high risk, the primary prevention strategies, and treatment of coronary occlusion will be discussed.

## Introduction

In the last two decades, there has been a trend toward a greater prevalence of bioprosthetic heart valves use even in younger patients (1–3). Recently, transcatheter aortic valve replacement (TAVR) has emerged as a non-inferior or superior alternative to medical treatment or surgical aortic valve replacement (SAVR) in high, intermediate and low-risk patients when transfemoral approach is available (4). All surgical bioprostheses have limited durability and usually fail within 10–15 years (5, 6). Similarly, transcatheter heart valve (THV) durability up to 6 years is already a well-established reality, with low rates of structural valve dysfunction (SVD) demonstrated in large and methodologically rigorous studies (2). However, data on clinical outcomes and THV integrity after 6 years remain very scarce. Increased life expectancy and the use of the bioprosthetic valve in younger patients led to an increased incidence of bioprosthetic valve failure (7).

In this context, implantation of a THV inside the failed aortic bioprosthetic valve (valve-in-valve, ViV) has emerged as an effective and less invasive treatment for degenerated aortic bioprostheses (8). Although procedural success is achieved in the great majority of patients, this procedure is associated with several potential complications (9), being coronary occlusion one of the most impactful on acute patients' prognosis (10). In this review, the mechanisms, the identification of patients at high risk, the primary prevention strategies used during pre-TAVR ViV screening and during the procedure itself to avoid coronary occlusion will be discussed.

## Incidence, Mechanism, and Risk Assessment of Coronary Occlusion after TAVR-ViV

Coronary occlusion is a rare complication after TAVR-ViV procedures, but it is associated with a very high mortality rate (9). It is four- to six-fold more common in ViV procedures than TAVR in native valves, and a higher risk of delayed coronary occlusion has been also reported with self-expanding TAVR devices (11).To date, the incidence of coronary occlusion during TAVR is 0.5% in the Transcatheter Valve Therapy Registry data and 2.3% in the Valve-in-Valve International Data registry, with related in-hospital mortality of ~50% (12, 13). However, this phenomenon could be even underestimated because it can be incomplete and not well-recognized.

When coronary occlusion does occur, the clinical presentation is usually characterized by ST-segment changes, severe hypotension and procedural ventricular arrhythmias that may require temporary cardiopulmonary support and revascularization (14, 15). Similar to TAVR in native aortic stenosis, meticulous pre-procedural planning is necessary to avoiding peri and post-procedural complications. Assessing the risk of coronary occlusion requires a deep understanding of the involved mechanisms. In TAVR-ViV procedures, coronary occlusion usually occurs when a bioprosthetic leaflet comes in contact with the coronary ostia or when the bioprosthetic valve contacts the aortic wall above a coronary ostium at the level of the aortic root as the positioned THV creates a covered cylinder in the root or the aorta (16). This is common to all THV and is correlated with the different characteristics of surgical heart valve (SHV) and the relationship of its bioprosthetic leaflets with the coronary ostia.

A recent study evaluating the safety and efficacy of TAVR-ViV for stentless bioprosthetic aortic valves showed that subcoronary implant technique, short simulated radial valve-to-coronary distance and low coronary height are predictors of coronary occlusion (17).

**Figure 1:**
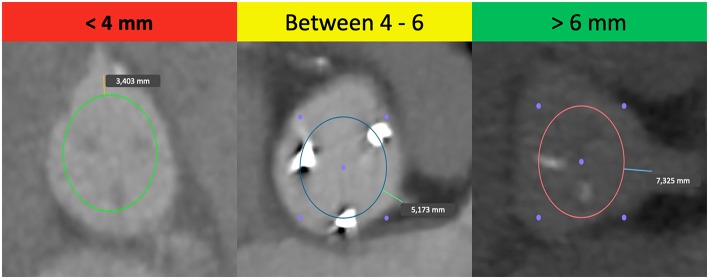
Examples of the “virtual THV to coronary artery” distance (VTC) in high-risk, borderline and low-risk patients for coronary occlusion in TAVR-ViV procedure.

Considering all these aspects, it is crucial to know the patient's anatomy before a ViV. The main predisposing factor in ViV procedures is the proximity of a coronary ostium to the anticipated final position of the displaced bioprosthetic leaflets after the new THV implantation (18), calculated by the virtual THV to coronary distance (VTC). This is a CT-obtained predictor of the proximity of the coronary ostia to the anticipated final position of the displaced bioprosthetic leaflets after THV implantation. It added the THV size to the classical risk factors of coronary ostia height and sinus width, taking into account also the THV tilting into the aortic annulus (16). To calculate the VTC, it is first necessary to identify the basal ring plane and the geometric center of the SHV. Then, a virtual cylinder that represents the THV is placed and aligned in the middle of the basal ring. At the end, the horizontal distance between the ostia of the coronary arteries and the edge of this cylinder is measured with a tool of the CT imaging software (16). If it is <4 mm the risk is maximum, between 4 and 6 mm is borderline and >6 mm is a low risk for coronary occlusion (Figure 1) (16). Furthermore, others possible risk factors for coronary occlusion after a ViV implantation may be determined by anatomic factors such as narrow sinuses of Valsalva and sinotubular junction, bioprosthetic valve factors such as supra-annular position, high leaflet profile, THV factors such as extended sealing cuff of high THV implantation, and finally internal stent frame (e.g., Mitroflow, Trifecta) or no stent frame (homograft, stentless valve) and bulky leaflets (19).

## THV Selection in High-Risk Patients

A meticulous understanding of the differences of SHVs design is important to allow the optimal selection of THV and to prevent coronary occlusion.

**Figure 2:**
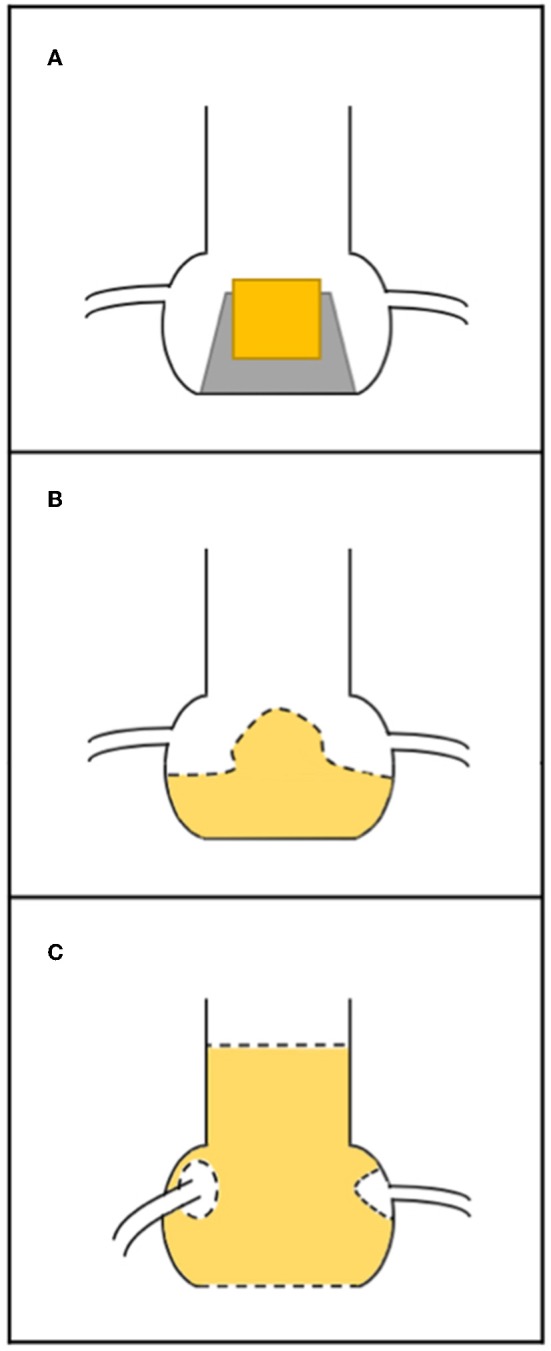
Relationship between surgical bioprosthesis and the coronary ostia. **(A)** Stented valve; **(B)** stentless valve replaced as subcoronary implantation (the dashed line represents the suture); **(C)** stentless valve replaced as full root, with suture line (dashed line) at the level of annulus and the coronary ostia have been reimplanted higher up.

Coronary occlusion may be more common in stentless bioprosthetic valves or those that are internally stented (e.g., Mitroflow or Trifecta) because the leaflets of these bioprostheses may extend outward in tubular fashion after a ViV procedure beyond the surgical device frame (10). Furthermore, it is crucial to know the nature of surgical implantation. In the subcoronary technique, the risk of coronary obstruction is greater than in full root replacement, because the suture line between the stentless prosthesis and the aorta is closer to the native coronary ostia (Figure 2) (20). In the case of stented SHVs, it is important to consider the “true internal diameter” for the choice of THV size and type.

Sizing, position and type of implanted THV may influence the risk of coronary occlusion after ViV. The selection of a small diameter THVs or intentionally under-expanded balloon-expandable THVs may result in less lateral displacement of surgical valve leaflets and posts with a minor risk of coronary occlusion after ViV (21).

Accurate positioning of the THV is essential for achieving good procedural results. The recently developed Valve in Valve Aortic App provides a very useful guide for THV positioning in available bioprostheses (22). In patient with high risk of coronary occlusion, a lower positioning of THV within the bioprostheses may cause less outward displacement of the surgical valve leaflets and posts than a THV implanted high.

The choice of the THV type for ViV-TAVR procedures should be individualized for each patient. The assessment of the risk for coronary occlusion may indeed influence THV selection. THV device that could be immediately retrieved after partial device implantation is advantageous (e.g., Lotus, Portico, Evolut-R, etc.). Furthermore, new-generation, fully retrievable THV devices or those with aortic leaflet clipping (JenaValve; JenaValve Technology GmbH, Munich, Germany) may be preferable if the risk of coronary occlusion is estimated to be high (23). The benefit of using devices with clipping mechanism in high-risk cases for coronary occlusion should be studied further.

## Coronary Protection

**Figure 3:**
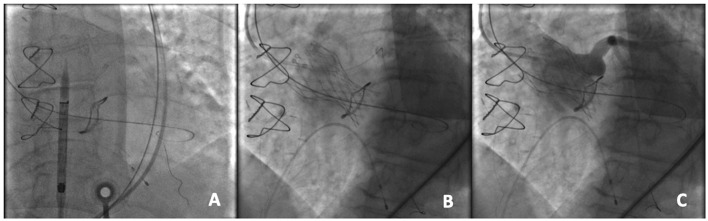
An example of coronary protection during a TAVR-ViV procedure. **(A)** Placement of the coronary guidewire in the left anterior descending (LAD) and A 5.5 × 15 mm undeployed balloon in the proximal LAD prior to the transcatheter aortic ViV implantation; **(B)** Deployment of the CoreValve Evolut PRO 23 mm device within a Mitroflow 21 mm; **(C)** Aortography to demonstrate the patency of the coronary ostium.

The management in patients with a coronary occlusion by a displaced bioprosthetic valve leaflet after ViV represents a challenge because they are commonly unstable and delivery of wire and successively a stent toward the coronary vasculature may be very challenging. In patients at high risk of coronary occlusion, it is essential to implement invasive primary prevention strategies, such as coronary protection with a supportive coronary guidewire (BHW, Abbott, Vascular) and an undeployed balloon or stent in the periphery of the left anterior descending in order to be ready to inflate the balloon or to place the stent at the coronary ostium to maintain coronary flow in case of its impairment (Figure 3) (19, 24). If the patency of the coronary cannot be restored and the hemodynamic is poor, the valve should instantly be snared (MCV), or removed from its anatomical position by using an oversized balloon (i.e., ESV prosthesis) and pulled up out into the ascending aorta to maintain coronary flow (18). However, ostial stenting after a leaflet obstruction is related with delayed coronary occlusion, high restenosis risk, difficulty re-engaging the coronary ostia, and difficulty retrieving undeployed stents (19, 25).

## Chimney Technique

In the majority of the cases of coronary occlusion after VIV procedure published in the past, the only solution was immediate cardiac surgery to perform an aortocoronary bypass. In case of coronary occlusion a less invasive option to solve it could be PCI with a stent to get enough radial force to maintain coronary flow. Chimney technique has recently described for coronary stenting in patients at high risk of coronary occlusion during ViV (26, 27). Originally, this technique was described for renal and mesenteric preservation after endovascular aortic repair with an excellent outcome for the patient (28). It involves deployment of the stent into the coronary ostia, with the proximal parts placed between the aortic wall and the bioprosthetic leaflets, and extended beyond coronary ostia to ensure coronary flow.

It has been also previously reported in limited case reports in prophylactic setting (29, 30). The prophylactic Chimney technique offers a potential predictable stepwise method of coronary protection that may be employed in the highest coronary occlusion risk patients. Long-term outcome data from this technique are expected to give a perspective of the long-term durability of this technique.

## Basilica

**Figure 4:**
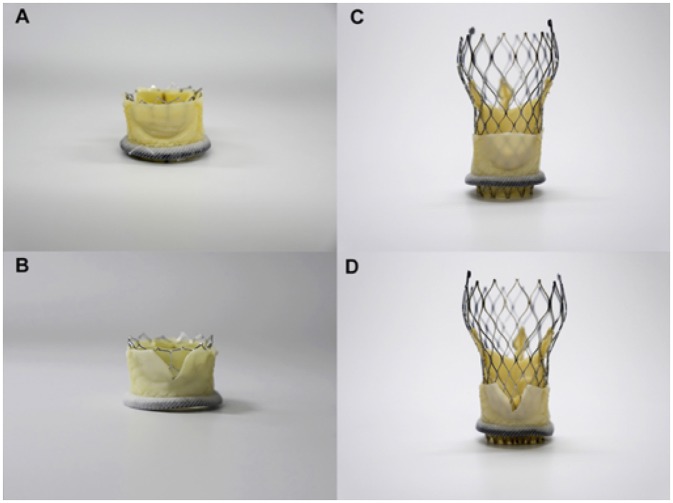
Simulation of BASILICA: Two different transcatheter heart valves [23-mm Sapien 3, **(A,B)**; 26-mm Evolut Pro **(C,D)**] implanted in a 25-mm Mitroflow; **(A,B)** Without BASILICA, **(C,D)** With BASILICA.

The bioprosthetic aortic scallop intentional laceration to prevent iatrogenic coronary artery occlusion (BASILICA) procedure has recently emerged as a method for disrupting bioprosthetic leaflets in patients at high risk of coronary occlusion. This procedure is based on the LAMPOON procedure, which uses catheters to split the mitral valve leaflet to prevent the obstruction of the left ventricular outflow tract (LVOT) after transcatheter mitral valve replacement (31, 32). The aim is to create a triangular space that allows blood flow into the coronary artery. The BASILICA procedure has three steps: First, under fluoroscopic and/or echocardiographic guidance, a multipurpose guiding catheter with a combination of 300 cm 0.014″ guidewire and microcatheter is advanced to the coronary cusp targeted for laceration, and a snare catheter is positioned in the left ventricular outflow; Next, the cusp is penetrated with an electrified wire and it is snare-retrieved and externalized; Then, the aortic valve leaflet is lacerated with electrosurgery energy (typically 70 W) in short bursts, until it is complete and the guidewire is free. In this way, a triangular space that allows blood flow is created (Figure 4). Finally, a conventional TAVR is performed (33, 34). Prophylactically coronary artery stent systems and cracking of failed bioprosthetic heart valve frame, with a high-pressure balloon, may be used at operator discretion (34). Currently, BASILICA is being prospectively evaluated in a clinical trial (NCT03381989) and further data are awaited on this promising technique.

## Conclusions

Coronary occlusion remains one of the major concerns of transcatheter aortic Valve-in-Valve implantation. Despite its low frequency, it is related to very poor prognosis. To avoid this complication, meticulous procedural planning is necessary to choose the correct prosthesis type and size, because this concern is universal to all THV designs. In this context, coronary protection should be used in high-risk cases to restore instantly coronary flow and improve clinical outcome. The Chimney technique has recently described for coronary stenting in patients at high risk of coronary occlusion during ViV. Furthermore, the bioprosthetic aortic scallop intentional laceration to prevent iatrogenic coronary artery obstruction (BASILICA) procedure is emerging as an effective method for preventing coronary occlusion. We can expect that new tools and techniques to treat failed bioprosthetic valves will continue to be designed.

## Author Contributions

RV and GC provided the first revision of the manuscript. MB made critical revisions of the text and gave final approval.

### Conflict of Interest

MB is consultant for Edwards Lifesciences, and an advisory board member for Biotronik. The remaining authors declare that the research was conducted in the absence of any commercial or financial relationships that could be construed as a potential conflict of interest.
